# A Large, Diverse, Urban Cardiovascular Health e-Cohort in Childhood and Adolescence: Protocol for the Young Hearts Study

**DOI:** 10.2196/82619

**Published:** 2026-05-21

**Authors:** Lucia C Petito, Rachel Zmora, Aashima Chopra, Yaojie Wang, Darci Phillips, Ileah Rios, Mark Pletcher, Rupali Gandhi, Huma Khan, Cheryl Lefaiver, Amanda Luff, Rashmi Narayan, Sarah De Ferranti, Holly C Gooding, Stephen Daniels, Brad Appelhans, Karen Lui, Francis J Alenghat, Rachel Caskey, Matthew M Davis, Marc B Rosenman, Darwin R Labarthe, Donald M Lloyd-Jones, Amanda M Perak, Norrina B Allen

**Affiliations:** 1 Department of Preventive Medicine, Northwestern University Feinberg School of Medicine, Ste 1400, 680 N Lake Shore Dr, Chicago, IL, 60611, United States, 1 3129087914; 2 Department of Epidemiology and Biostatistics, School of Medicine, University of California, San Francisco, Francisco, CA, United States; 3 Advocate Children's Medical Group Pediatric Cardiology, Advocate Health Care, Oak Lawn, IL, United States; 4 Advocate Children's Medical Group Adolescent Medicine, Advocate Health Care, Evergreen Park, IL, United States; 5 Advocate Aurora Research Institute, Advocate Health, Milwaukee, WI, United States; 6 AllianceChicago, Chicago, IL, United States; 7 Department of Cardiology, Boston Children’s Hospital, Boston, MA, United States; 8 Department of Pediatrics, Harvard Medical School, Boston, MA, United States; 9 Department of Pediatrics, Emory University School of Medicine and Children’s Healthcare of Atlanta, Atlanta, GA, United States; 10 Department of Pediatrics, University of Colorado Anschutz Medical Campus And Children’s Hospital Colorado, Aurora, CO, United States; 11 Department of Family and Preventive Medicine, Rush University System for Health, Chicago, IL, United States; 12 Department of Medicine, University of Illinois Chicago, Chicago, IL, United States; 13 Department of Medical Social Science, Nemours Children's Health, Jacksonville, FL, United States; 14 Stanley Manne Children’s Research Institute, Chicago, IL, United States; 15 Department of Pediatrics, Northwestern University Feinberg School of Medicine, Chicago, IL, United States; 16 Mary Ann and J. Milburn Smith Child Health Outcomes, Research, and Evaluation Center, Ann and Robert H. Lurie Children’s Hospital of Chicago, Chicago, IL, United States; 17 Boston University Chobanian & Avedisian School of Medicine, Boston, MA, United States; 18 Department of Pediatrics, Division of Cardiology, Northwestern University Feinberg School of Medicine, Chicago, IL, United States; 19 Ann and Robert H. Lurie Children’s Hospital of Chicago, Chicago, IL, United States

**Keywords:** e-Cohort, prospective study, cardiovascular health, pediatrics, health behaviors

## Abstract

**Background:**

Cardiovascular health (CVH), defined as a composite of 8 health factors and behaviors ranging from body composition to sleep duration, is strongly associated with the risk for future cardiovascular events in adults. However, there is little data on CVH among contemporary, diverse cohorts of children and adolescents.

**Objective:**

This protocol describes the completed recruitment of a diverse pediatric e-cohort based in Chicago. We include details about ongoing longitudinal data collection for CVH behaviors, as well as neighborhood and environment-based socioeconomic factors, and linkage to electronic health records (EHRs) for direct capture of CVH factors.

**Methods:**

Young Hearts (YH) uses a longitudinal cohort design, linking participant responses to annual electronic surveys to EHRs. Parents/guardians of children aged 0-17 years and adolescents aged 18 years are asked to complete surveys at 3 e-visits, completed annually in consecutive years. Adolescents aged 12-17 years are invited to complete surveys at each e-visit to provide self-reports of their data in addition to their parents/guardians. As adolescents reach the age of majority, they re-consent and complete their remaining e-visit data. Participants’ clinical data are abstracted from EHRs at 6 health systems in Chicago and linked to their survey data. The main study outcome is the CVH score, adapted from the Life’s Essential Eight framework for use in pediatric populations. We will also study component CVH behaviors and factors, each assessed using developmentally-appropriate measures, including body mass index, blood pressure, lipids, glycemic control, diet, physical activity, sleep, and smoking exposure. Our innovative study design will allow us to model trajectories of pediatric CVH from birth through 20 years of age. Funding for this study was provided by the National Heart, Lung, and Blood Institute (R01HL155864).

**Results:**

The YH study, funded in March 2021, recruited, enrolled, and consented 7114 children and adolescents aged 0‐18 years between April 2022 and February 2025. Baseline surveys have been completed by 6651 participants, of whom 3259 (49%) were female, and the mean age was 7.4 (SD 5.4) years. Data collection is still in progress, with completion anticipated in February 2027. We aim to publish results beginning in 2026.

**Conclusions:**

This study will define the epidemiology of cardiovascular risk factors in a diverse, contemporary cohort of US children and adolescents and identify behavioral and structural individual- and area-level economic, psychosocial, and lifestyle factors associated with identified disparities. We anticipate that the YH cohort will become an ongoing resource for pediatric CVH research.

## Introduction

### Background

The American Heart Association defines cardiovascular health (CVH) using “Life’s Essential Eight” (LE8) behaviors and factors, including diet, physical activity, sleep, nicotine exposure, BMI, blood pressure (BP), lipids, and glucose [1]. In adults, high CVH, that is, favorable levels of all 8 CVH behaviors and factors, is associated with a multitude of benefits, including a decreased incidence of cardiovascular (CV) diseases, cancer, depression, and dementia, as well as a longer, healthier lifespan [2]. However, less than 5%‐10% of the adult US population has high CVH, and its prevalence decreases with age; by age 8 years, over 20% of children no longer have high CVH [3,4]. Accumulation of nonoptimal CVH factors and behaviors early in life translates to greater CV risk and burden of CV disease later in life.

The current generation of children is the first in centuries to have a shorter projected lifespan than their parents [5,6]. One of the main drivers of this decline in health is the growing prevalence of nonideal CV risk factors and behaviors, particularly obesity, among youth. While obesity has been well-studied in childhood, the advantage of examining CVH is that it includes both the behaviors that contribute to adiposity as well as the clinical consequences, including dyslipidemia, hypertension, and diabetes that may stem from it. CVH provides a summary of the global burden of chronic disease risk in an understandable framework for patients and actionable points of intervention in clinical care.

Current definitions and measurement methods for CVH in youth are limited because of measurement burden and uncertain applicability across developmental transitions. For example, completing a gold standard dietary assessment with two 24-hour dietary recalls can take more than 60 minutes and is infeasible in clinical practice. Additionally, although there are clinical guidelines to define “abnormal” levels of clinical metrics such as non-HDL cholesterol, these do not address the potential risk associated with levels that do not meet this threshold but are quite high compared with those of age-matched peers and may portend risk. Evidence is needed to develop and refine developmentally appropriate and clinically feasible ways of measuring CVH, particularly in infancy and early childhood [2].

Comprehensive measurement of CVH and its determinants in children demands rigorous collection of clinical, behavioral, and psychosocial data—a combination previously unavailable in any contemporary, diverse pediatric cohort. Established pediatric studies such as the US’s Environmental Influences on Child Health Outcomes cohort, the Young Finns Study, and Australia’s Childhood Determinants of Adult Health study have served as benchmarks in the field. However, these cohorts have not collected data on modern contextual factors contemporaneously with CVH; for example, the Young Finns and CDAH began data collection over 30 years ago [7,8], prior to the widespread use of cellphones. Although the Environmental Influences on Child Health Outcomes cohort features a large, contemporary sample with detailed socioenvironmental assessments, its comprehensive CVH data collection is restricted to BMI, while critical factors such as lipids and glucose are only available in a subcohort [9].

These cohorts and others have contributed valuable data to the International Childhood Cardiovascular Cohort (i3C) Consortium [10]. The i3C Consortium has provided the first evidence that childhood cardiovascular risk factors correlate with subclinical cardiovascular disease in early adulthood [11,12]. Subsequent analysis of i3C found that cardiovascular risk factors in childhood acted on adult cardiovascular disease both indirectly and directly [13], providing a compelling rationale for additional research. The current cohort was therefore established to address significant gaps in the literature and enhance our understanding of CVH and related social and environmental risk factors in youth populations.

### Objectives

The overarching goal of the Young Hearts (YH) study is to identify trajectories of CVH and its component factors and behaviors from birth through age 20 years among a large, diverse cohort of children and their families. Using an innovative study design for a pragmatic cohort using technology-enabled data collection, we plan to recruit children (or their guardians) from 6 city-wide health systems in Chicago. We will use 2 vehicles to collect data across all 8 components of the American Heart Association’s LE8: we will capture CVH behaviors via detailed e-surveys, and we will link to available clinical CVH factors in the electronic health record (EHR) of participating health systems. All participants will be followed longitudinally for 2 years to better understand the changing trajectories of pediatric and adolescent CVH. Specifically, we plan to accomplish the following specific aims.

First, we plan to identify trajectories of CVH and its component factors and behaviors from birth to young adulthood within a large, diverse cohort of participants (target enrollment >7000) across the city of Chicago. Then, we will identify predictors of CVH trajectories, including a broad range of markers of resilience and vulnerability, including sociodemographic characteristics, economic stability, residential and neighborhood environment, social context and support, neuropsychological skills, parental CVH, and family health history. Last, we will develop an accurate and generalizable CVH growth curve for use in clinical care to target prevention efforts to children at high risk of declining CVH and subsequent CVD.

## Methods

### Overview

The YH study is being conducted by a team of researchers at Northwestern University, in partnership with 7 health systems across Chicago, IL, USA. Children (or legal guardians of children) ages 0 to 18 years will be recruited from across Chicago. Participants will be asked to complete a developmentally appropriate and clinically feasible set of online surveys and questionnaires at 3 e-visits, spaced approximately 12 months apart, for a total of approximately 2 years of follow-up. These surveys and questionnaires will cover CVH behaviors, as well as markers of resilience and vulnerability. EHRs will be used to identify recent measurements of cardiovascular risk factors and related comorbidities, including BP, lipid levels, and familial hypocholesterolemia; these will be linked to their survey responses. The CVH behaviors and factors will be combined to calculate the primary CVH measure and age- and sex-specific CVH z-scores. Leveraging the repeated outcome assessments, additional analyses will examine within-child changes in CVH z-scores, behaviors, and factors over the 2-year period.

### Ethical Considerations

This is a fully remote study—there will be no in-person contact of study staff with participants. Northwestern University’s Institutional Review Board (IRB) approved the study protocol. Data use agreements for EHR data transfers were set up with each of the 7 participating health systems, Northwestern Medicine, Ann & Robert H Lurie Children’s Hospital of Chicago (Lurie), AllianceChicago (Alliance), Advocate Aurora Health, Rush University Medical Center (Rush), University of Illinois, and University of Chicago (UChicago); each health system’s IRB reviewed and approved the study protocol for site-specific distribution of study materials to eligible patients. Researchers interested in collaborating with YH or proposing an ancillary study should contact the corresponding author.

All children aged 0 to 18 years will be eligible to enroll in the study. Eighteen-year-olds, as legal adults, will provide their informed consent electronically. Each child aged 0 to 17 years will need a parent or legal guardian (hereafter “guardian”) to participate with them; informed consent for minors will be provided electronically by guardians, as well as assent from participants aged 12‐17 years. As participating children aged 16 and 17 years turn 18 years old before their second or third e-visit and gain legal independence, we will obtain electronic informed consent forms from them directly; the same applies to the collection of assent from pre-teens who turn 12 during the course of the study. At enrollment of their first participating child, guardians will be given the option to enroll additional children; this will facilitate future sibling studies.

Recruited participants (and guardians) will provide informed consent for linkage to their EHR. A unique study ID will be used to identify EHRs for YH participants. These IDs will ensure that no identifiers are shared between institutions and that all study data is not identifiable. All data will be recorded and stored in HIPAA-compliant, password-protected databases that can only be accessed by approved study personnel. No protected health information will be shared with employers, insurers, or nonresearch personnel.

Participants in the study will be provided with a US $20 incentive for each completed e-visit, for a maximum total compensation of US $60 per child. Compensation will be distributed via gift card, using the Hyperwallet (pre-2023) or Amazon (2023 onwards) platforms. Additionally, all participants who complete their baseline e-visit are entered into a raffle to win a US $150 Amazon gift card. The raffle will be conducted for every 1000 participants who complete their baseline e-visit until the end of recruitment. Each participant is included in exactly one drawing. These raffles will continue through the follow-up surveys, with one raffle occurring for every 1000 participants who complete their 1st and 2nd follow-up surveys.

### Study Settings and Participant Recruitment

We are working with several partners, including the 7 health systems listed above, to achieve our recruitment goal of at least 7000 children aged 0 to 18 years from across Chicago. Our recruitment efforts will be divided into 2 strategies: population-based and site-based recruitment.

The population-based recruitment strategy will use targeted approaches in specific neighborhoods across Chicago to engage potential participants. This includes heavy placement of advertisements in areas with high concentrations of targeted socioeconomic, race, and ethnic groups. Additionally, we will partner with community organizations and other studies to distribute brochures. For instance, we will collaborate with the Patrick M. Magoon Institute for Healthy Communities, providing them with flyers for distribution. Of these, the majority will be given to their Mobile Health Program, which offers medical services in underserved neighborhoods throughout Chicago. The remaining flyers will be given to their volunteer coordinator to display at food pantries and other community events. Other efforts will include coordinating with leadership and staff at the Northwestern branch of the All of Us Research Program, a nationwide research program to advance precision medicine, and the Early Start program, a nonprofit organization promoting child development in Illinois, to distribute flyers to promote participation in YH.

This study will involve the linkage of participant survey data with existing EHRs, so we will collaborate with participating health systems to conduct site-based recruitment. This strategy, recruiting current patients at each site to participate in YH, will maximize data availability on CV factors. The mode of recruitment at each site will vary, but is primarily electronic, including emails, SMS texting, and MyChart messages; a small number of postcards will be sent as well. Each site will send 2 or 3 additional electronic reminders after their initial contact, inviting participation in YH, by either email, SMS messaging, or MyChart messages.

### Study Protocol

**Figure 1.**
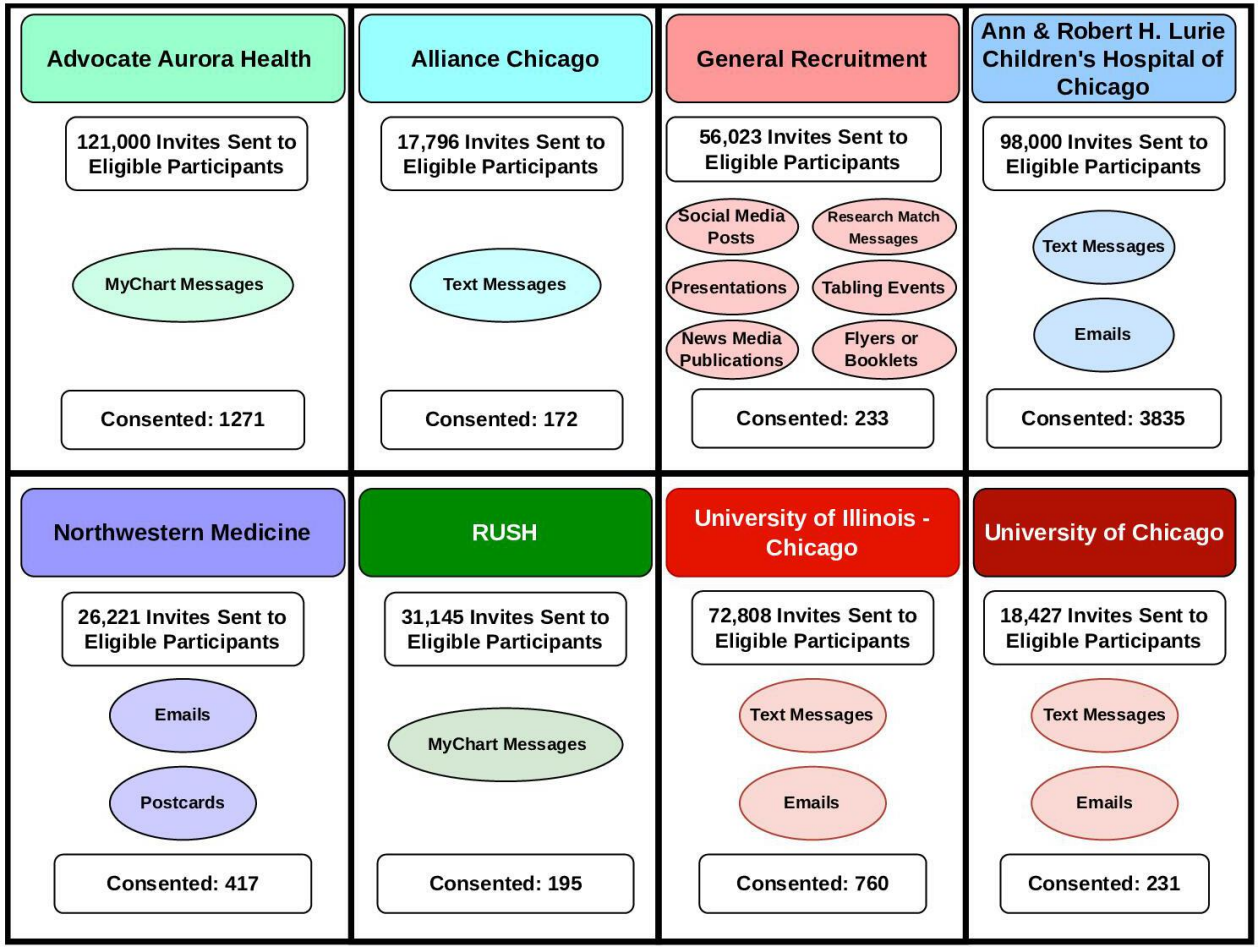
Summary of recruitment efforts and consent of 7114 children and adolescents into the Young Hearts cohort.

The YH study will be a longitudinal e-cohort study of children and adolescents in the Chicagoland area. After recruitment and consent, participants will complete electronic surveys at 3 e-visits (baseline and 1 and 2 years after baseline), each of which takes approximately 30 min to complete via the web-based portal through the Eureka Research Platform (University of California, San Francisco) [14]. Participants aged 18 years and older will complete all surveys about themselves. Participants aged 12‐17 years, once assented, will be asked to complete a set of age-appropriate surveys. Participants aged 0‐17 years will have guardians fill out surveys on their behalf. EHRs available from 7 participating health systems will be linked to participant survey responses. Figure 1 describes the recruitment of participants by site. Data collected in surveys and from the electronic medical record are described below.

Each e-visit consists of answering 6 questionnaires in total; all questions must be answered to open subsequent questionnaires. If any questionnaires are incomplete, the web app sends reminders on days 3, 7, 14, and 30 via email, text if a phone number exists with SMS capabilities, and notifications through the mobile app (only for participants who download the app). Although participation in YH is done fully remotely, study staff will be accessible by phone or email to all potential and consented participants during regular work hours, Monday through Friday from 9 AM to 5 PM. Recruitment messages sent to eligible patients will include the shared study email and a direct phone number for a designated staff member.

### e-Survey Data Collection via the Eureka Mobile Research Platform

The Eureka Research Platform was used to enroll, obtain consent, and collect survey data. Eureka is a National Institutes of Health–funded (5U2CEB021881) direct-to-participant eConsent and data collection system. The Platform uses a HIPAA-compliant design template and technology developed in the Health eHeart Study that has been used for web-based recruitment of over 260,000 consented research participants [15]. Additional information about the platform is available [14,16].

### Retention Strategies

Once participants have completed their consent forms, Eureka sends reminders for them to complete their first e-visit (baseline surveys) 4 times in the first 30 days. These reminders are also scheduled for the 2nd and 3rd e-visits (1 and 2 years post-baseline). After the initial 30-day period, study staff will contact participants who have not completed their e-visits via text, email, or phone calls. These contacts notify participants that the e-visits are incomplete and remind them of the financial incentive for completing them.

To encourage participant engagement between e-visits, the study staff, together with input from the study investigators, write newsletters about the scientific content of the study. For example, the first newsletter introduced study participants to the concept of CVH scores and their components. Other topics for past newsletters include sleep, healthy eating, and physical activity. Future topics will cover physical activity and body composition, BP, cholesterol, and blood glucose. These newsletters are written for lay audiences and emphasize the role of each topic within childhood and adolescence, providing targeted content that is age-appropriate. In addition, after the initial study visit, participants and their guardians are notified via email of their child’s component LE8 behavioral CVH scores (diet, physical activity, sleep, and nicotine exposure).

### Community Engagement

The YH Community Advisory Board (CAB) was established in May 2023. Recruitment efforts targeted professionals working with young children via LinkedIn messages and guardians of YH participants through email outreach. Ensuring diversity within the CAB is critical, as it brings varied insights and perspectives that enhance decision-making and maximize the study’s impact. As such, guardians were randomly selected for CAB membership based on criteria including race, income, gender, and country of origin. The CAB has provided critical feedback on key areas such as survey enhancement, recruitment strategies, retention efforts, community education activities, and the dissemination of study results. Notably implemented suggestions from the CAB include more comprehensive study descriptions and reassurance of incentives prior to surveys, incorporating graphics and engaging images in youth surveys, and strategically placing flyers in key community locations to boost cohort diversity.

To address the survey response rates among guardians of our younger participants, we collaborated with Northwestern’s Center for Community Health in hosting a one-time Youth Shared Resource Panel. Our Shared Resource Panel was comprised of 8 members, aged 14‐17 years old. The youth panel provided specific feedback on how to increase survey participation among minors, who respond to surveys at a lower rate than their guardians. Two key suggestions emerged from the panel: first, improving the youths’ understanding of the importance of the research and CVH before asking that they complete the surveys; and second, requiring guardians to enter their children’s cellphone numbers so that survey notifications are sent via SMS rather than email.

### Measures

#### e-Survey Questionnaires

This survey was created using questions from published instruments to briefly assess each domain of interest (Table 1). Survey length was prioritized to minimize participant burden. All questionnaires were designed to be developmentally appropriate; multiple versions were created to be administered to, for example, infants versus teenagers. The full questionnaires are available upon request.

**Table 1.** Schedule of survey modules for the young hearts study.

Survey components	Parent/guardian	Adult child (18+ years)	Minor child (12-17 years)
Demographics			
Parent			
Relationship with child	✓^a^		
Race and ethnicity	✓^a^		
Nativity	✓^a^		
Education, employment, income	✓		
Marital status	✓		
Child			
Birthdate	✓^a^	✓^a^	
Sex and gender	✓	✓	✓
Race and ethnicity	✓^a^	✓^a^	
Nativity		✓^a^	
Education	✓	✓	
Employment, household income		✓	
Marital status		✓	
Health care coverage	✓	✓	
Family			
Household members	✓	✓	
Living situation	✓	✓	
Neighborhood questionnaire	✓	✓	
Six-Item Food Security Scale	✓	✓	
Financial strain	✓	✓	
Medical history			
Height and weight	✓	✓	
Family medical history	✓	✓	
Health status	✓	✓	
Pregnancy and birth complications	✓^a^	✓^a^	
Developmental milestones	✓	✓^a^	
Health care utilization	✓	✓	
Medication	✓	✓	
Pregnancy history		✓	✓
Social context and support			
Strengths and Difficulties Questionnaire (SDQ)	✓^b^	✓	✓
Weekly activities	✓^b^	✓	✓
Perceived Stress Scale (PSS-4)	✓	✓	✓
Patient Health Questionnaire (PHQ-2)	✓	✓	✓
School experiences		✓	✓
Lifestyle and behaviors			
Diet (NHANES^c^)	✓^b^	✓	✓
Diet Behavior and Nutrition (DBQ)	✓^a^		
Comprehensive Feeding Practices Questionnaire	✓		
Emotional Eating Scale		✓	✓
Physical activity	✓	✓	✓
Sleep	✓	✓	✓
Family routines	✓	✓	✓
Secondhand smoke exposure	✓	✓	✓
Smoking (NHANES)		✓	✓
Screentime	✓	✓	✓
Alcohol and marijuana		✓	✓
Childhood experiences			
Adverse Childhood Experiences Questionnaire (ACE-Q)	✓	✓	✓

a Survey only administered at the baseline e-visit.

b Survey only administered at e-visits when participants were aged 2 years or older.

c NHANES: National Health and Nutrition Examination Survey.

#### Cardiovascular Health Behaviors

Data are collected on the following 4 domains. These data are scored (range 0‐100) using the LE8 Framework [1]; the average of these 4 scores is used as a composite score of behavioral CVH. Details surrounding the scoring of each component are available in Table S1 in Multimedia Appendix 1.

- Diet: Diet quality is assessed using 2 questions in infants (0‐1 y) and 6 questions in older youth (2‐20 y) [17,18]. For infants, questions address breastmilk or formula feeding and the introduction of other foods. For older youth, questions address intake of fruits, vegetables, sugar-sweetened beverages, fast food, salty discretionary foods (eg, potato chips), and sweet discretionary foods (eg, cake). These questions, slightly modified for best applicability to American children, have previously been shown to have good reproducibility and moderate to good validity against 24-hour dietary recalls in a sample of Australian children [18].
- Physical activity: Physical activity is assessed with 2 questions for each of 3 age groups: 0‐12 months, 1‐5 years, and 6‐20 years. For infants and young children (0‐12 mo and 1‐5 y), questions are based on physical activity standards from the American Society of Health and Physical Educators (SHAPE) and the American Academy of Pediatrics (AAP) [19]. These include time spent on a safe floor space for free movement and time in devices (eg, high chairs) that restrict movement (0‐1 y), or duration of structured (eg, games) and unstructured active play (1‐5 y). Questions for youth ages 6‐20 years address the number of days per week they are moderately-vigorously active for at least 60 minutes and the total duration of moderate-to-vigorous activity per week [20-22].
- Sleep: Sleep quantity, the target of pediatric sleep guidelines, is assessed with 6 questions for children aged 0‐5 years and 4 questions for ages 6‐20 years [23,24]. For all ages, questions assess usual bedtime and waketime on weekdays and weekends, and for ages 0‐5 years, additional questions address the number and duration of naps.
- Smoking: Current secondhand smoke exposure (number of people in the home and other settings who smoke, number of days child exposed) is assessed for all ages with 4 questions from the National Health and Nutrition Examination Survey [22]. We assess current cigarette and e-cigarette smoking (ever tried, quantity used in past 30 days) among youth aged 12‐20 years with 4 questions from the National Youth Tobacco Survey [25].

#### Self-Reported Medical History

The guardians of participants aged 0-17 years (or participants aged 18 years) are asked to report their child’s current general health status. They also provide information about developmental milestones and health history. They are also asked to provide family health history, such as familial hypercholesterolemia, diabetes, cancer, and heart disease.

- Height and weight: Current height and weight are reported by guardians for all youth.
- Health status: Guardians report their child’s health status as “excellent,” “very good,” “good,” “fair,” or “poor.” We also assess the presence of physical disabilities and chronic health conditions (eg, asthma) and age at the time of onset or diagnosis. Guardians also report the child’s hospitalization history, including the frequency, reason, and age at hospitalization.
- Medication: Guardians are asked to self-report the current medications their child is taking.
- Pregnancy: At baseline, guardians are asked if there were any complications during pregnancy or the birth of the child. Guardians are also asked about the delivery method, birth weight, prematurity, and weeks of gestation of the child.
- Development: Guardians complete several questions about developmental benchmarks at baseline, including the age when the child sat, walked, spoke, and was potty-trained. Guardians also report the presence of growth, motor, or speech concerns.
- Menarche: Adolescent girls aged 12‐17 years and their guardians report age at menarche. Adolescent girls are also asked if they have ever been pregnant or used birth control.
- Health care access: Guardians report the child’s primary point of care, the name and address of their clinic, and the name of their doctor.
- Family Health History: Guardians (or adult participants) complete a brief medical history, which includes the presence of chronic health conditions (eg, high BP) among immediate family members.

#### Risk and Resilience Factors

We assess risk and resilience factors, including social determinants of health, with attention to age-appropriate assessment (Table S2 in Multimedia Appendix 1). Inclusion of social determinants was informed by the Institute of Medicine’s recommended social domains for inclusion in EHRs, in addition to Healthy People 2020’s social determinants of health.

- Sociodemographic factors: Guardians report their child’s race, ethnicity, nativity, sex, gender, grade level, and health care coverage. In addition, guardians report their own race, ethnicity, educational attainment, employment status, household income, marital status, and nativity. Guardians also report their relationship to their child. Adolescents aged 12‐17 years report their sex and gender.
- Economic stability: Guardians report their educational attainment, employment status, household income, and household members. Guardians complete the Six-Item Food Security Scale, which characterizes food security as high or marginal, low, or very low food security [26]. Financial strain is assessed using a 6-item screener [27]. Guardians report the family supports, which they use, such as the Link Card (Illinois Supplemental Nutrition Assistance Program [SNAP] card) or Child Care Assistance Program. Guardians also report their living situation, including the type of housing and use of housing assistance.
- Neighborhood environment: Guardians complete an 11-item neighborhood environment questionnaire, which includes their impressions of neighborhood safety, social involvement, and public service domains [28]. We plan to geocode residential addresses in order to further explore the impact of neighborhood physical and social environments on childhood CVH; among others, this will include the Childhood Opportunity Index 3.0, which includes education, health and environment, and social and economic domains [29].
- Social context and support: Family routines, including eating and sleeping routines, are assessed at all ages. For infants, we assess the eating routine as the usual number of feedings provided within 24 hours. For all other ages (1‐20 y), eating routine questions include the number of meals and snacks per day, the number of times per week dinner is consumed with at least one other family member, and whether dinner is usually consumed in front of a screen [30]. For sleeping routines, questions address the maximum difference (in minutes or hours) between the earliest and latest bedtime and waketime during the week and on weekends, allowing capture of social jet lag [31,32]. Guardians and adolescents report routine weekday activities (ie, chores, schoolwork, video games). Adolescents aged 12‐17 years report their school experiences including their relationships with peers.
- Adverse childhood experiences (ACEs): ACEs are assessed using the 17-item Pediatric ACE and other Determinants of Health Questionnaire [33] as the total number of events experienced, reported by the guardian (ages 0‐17) and child (ages 12‐20). This method of indicating the total number of ACEs, rather than specific experiences, allows the collection of critical data while adhering to mandated reporting laws in this large study, where there is no face-to-face contact with participants.
- Screen time: Screen time, including time spent on TV, TV-connected devices (streaming, video game consoles, DVDs), computers/laptops (outside of homework), tablets, or mobile/smart phones, is reported by guardians and adolescents aged 12‐18 years. Questions address screen time per day on weekdays and weekends.
- Alcohol and marijuana use: Adolescents aged 12‐17 years are administered several questions assessing use, frequency, and age of first use of alcohol and marijuana. Binge drinking is assessed with one question.
- Neuropsychological factors: Perceived stress is measured among guardians using the 4-item Perceived Stress Scale [34,35] and among adolescents aged 12 to 17 years using the 4-item Perceived Stress Scale-C [36]. Depression is assessed among guardians and adolescents using the Patient Health Questionnaire-2 [37]. Childhood assets are measured using the Strengths and Difficulties Questionnaire, which is completed by guardians and adolescents [38]. Emotional feeding is assessed among guardians using questions drawn from subscales of the Comprehensive Feeding Practices Questionnaire [39]. Guardians and adolescents are administered a shortened version of the Emotional Eating Scale, comprised of questions from 3 subconstructs: anxiety, depression, and unsettled, as well as additional questions regarding their history of binge eating, overeating, and eating in the absence of hunger, which were drawn from Project Viva’s adolescent questionnaire (items B3-5) [40-42].

#### Electronic Health Record (EHR) Data

Most study participants are recruited from targeted recruitment done through the 7 participating health systems: Northwestern Medicine, Lurie, Alliance, Advocate Aurora Health, Rush, University of Illinois, and UChicago. For these participants, we will link EHRs from the site where they were recruited, but not across institutions. The limited number of participants recruited during general recruitment (All of Us, Headstart, research match) will not have EHR data available.

**Figure 2.**
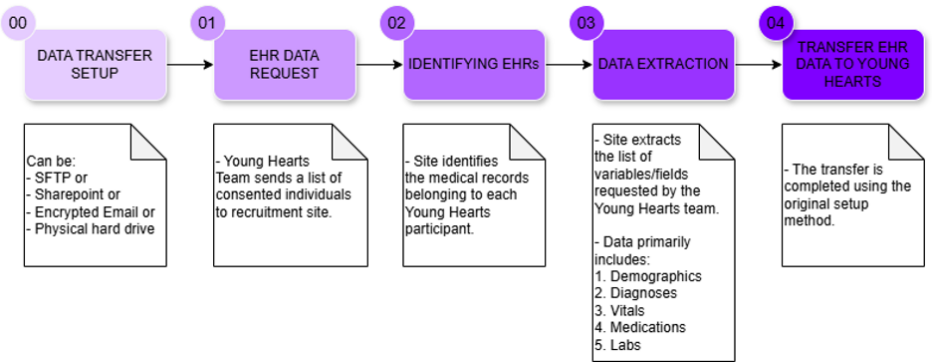
Linking electronic health records to data collected via surveys at e-visits: the young hearts study. EHR: electronic health record. SFTP: secure file transfer protocol.

Study staff will request the EHR data by securely sending each participating health system a list of consented participants with their unique codes. The health system will then abstract the requested records and send them to us using an IRB-approved file transfer protocol (Figure 2). This data pull will be done at least twice: once following the end of recruitment, and again roughly 2 years later, once all participants have filled out their e-visit 3 surveys. Other sites plan to do this data pull annually. The initial pull will cover all data available about consented participants, and the additional pulls will cover new encounters/visits of consented participants.

The EHR data will be reported across the following 6 domains: encounters [including type of encounter (eg, in-patient, ambulatory), payer (eg, Medicaid, self-pay, commercial)]; demographics (sex, age at time of encounter); diagnoses (ICD-10 codes); vitals (systolic and diastolic BP in mm Hg); laboratory values (high-density lipoprotein cholesterol, low-density lipoprotein cholesterol, total cholesterol, triglycerides, serum glucose, hemoglobin A1c, lead, C-reactive protein); and medications (anti-hypertensives, anti-diabetics, and lipid-lowering medications, as well as inhalers, ADHD, and birth control). Table 2 contains a summary of the requested relational databases.

**Table 2.** Description of relational databases containing electronic health records of Young Hearts participants from each participating health system.

Table	Description
Demographics	Demographic information from health system: birth year, sex, race, ethnicity, insurance
Encounters	Encounter ID, age at encounter, encounter type
Diagnoses	Encounter ID, diagnosis name, diagnosis code
Vitals	Encounter ID, vitals’ name, vitals’ value, vitals’ unit
Labs	Encounter ID, lab name, lab value, lab value units, lab code
Medications	All prescription orders: medication name, medication class, national drug code, start date, end date

#### Cardiovascular Health Factors

Clinical CVH factors, including BMI, BP, total cholesterol, and serum glucose, will be abstracted from the available EHR. Only measurements from ambulatory visits will be used.

- BMI: Weight and height (or recumbent length) are measured universally from birth to 20 years of age, and will be used to define BMI as weight in kilograms/height in meters squared. These will be further processed to sex-specific weight-for-length (age 0‐2) and BMI (age 2‐20 years) percentiles, including the extended BMI-for-age charts for those with BMI >97th percentile as recommended by the US Centers for Disease Control [43].
- BP: BP is measured universally beginning at the age of 3 years; measurements prior to that are taken if risk factors are present per NHLBI/AAP recommendations. These measurements will be translated into sex- and age-specific percentiles, incorporating the height of the child [44].
- Total cholesterol: Per NHLBI/AAP recommendations, cholesterol is measured universally once between ages 9‐11 years, and again at 17‐21 years. It is only measured at other ages if risk factors are present [45,46].
- Blood glucose: Per NHLBI and the American Diabetes Association, blood glucose is measured if a physician suspects type 1 diabetes or starting at age 10 years (or puberty) if risk factors for type 2 diabetes are present [47].
- Additional factors: To refine our definitions of the factors described above, we will use available encounter diagnosis codes to identify chronic conditions, such as obesity, diabetes, hypertension, and familial hypercholesterolemia.

### Combining EHR Data With e-Survey Data: Planned Data Processing

After the raw data have been processed as described above, the EHR data will be linked to the e-Survey data. For each component of clinical CVH, we will identify the measurement that took place closest in calendar time to the e-visit and record it as the YH measurement. All measurements of clinical CVH components will be available for study analyses as appropriate. This will facilitate our definition of CVH as it relies on using only the information gathered under current clinical guidelines (as detailed above) for our study to remain pragmatic and immediately clinically translatable.

For each of the health behavior and clinical age-specific CVH metrics, we will combine survey responses or data from EHRs to create one composite measure per metric that can be used to describe where a child falls relative to the population distribution at their current age. The age- and sex-specific population distributions are all assumed to be normal, so each child will be assigned to what amounts to a z-score and a percentile that corresponds to the age-specific population distribution. This idea has been detailed previously by our team [48]. These z-scores will incorporate all CVH factors and behaviors, providing a global measure of CVH in infants, children, and adolescents that can be used to study associations between CVH and outcomes across all ages. This z-score will be calculated based on the average score across all 8 components of CVH, with age-specific scoring detailed in Table S1 in Multimedia Appendix 1.

### Sample Size Considerations

As one of our goals is to create a reference standard for age-specific CVH z-scores, our sample size calculation centered around our ability to precisely estimate particular percentiles of a standard normal distribution. Thus, we chose to estimate the sample sizes necessary to precisely estimate the 50th percentile (median/mean), 16th percentile (1st SD), and 2.5th percentile (2nd SD) of a standard normal distribution at one particular age. Estimating these particular quantiles as accurately as possible should provide reassurance that 50% of children fall below the median, and that 68% and 95% of children fall within 1 and 2 SDs of the mean, respectively.

After investigating a range of sample sizes, we concluded that a sample size of 400 children at each age would give us sufficient precision to estimate the median, 16th percentile, and 2.5th percentile (Table S3 in Multimedia Appendix 1). Larger sample sizes are needed as the percentile nears 0, as we would expect fewer children to fall there. Therefore, we aimed to recruit a minimum of 7000 infants, children, and adolescents.

### Proposed Statistical Analyses

We plan to use generalized linear models to study the associations between novel cardiovascular risk and resilience factors and global metrics of pediatric CVH, including adapted Life’s Essential 8 scores (defined in Table S1 in Multimedia Appendix 1) and CVH z-scores. Initial analyses will use only data collected at the baseline visit, studying cross-sectional associations. Further analyses will use generalized mixed linear models using repeated measures of CVH z-scores on the same children, to create pediatric CVH growth curves showing the distribution of CVH z-scores over childhood. We will also use group-based trajectory modeling to identify children who have a similar underlying trajectory of CVH z-scores. Once children have been assigned to a trajectory group, we will use multinomial regression models to identify risk and resilience factors that are associated with group membership. Age-specific multiple imputation (M=50) of missing data is planned for all analyses; Rubin’s rules will be used to combine estimates across datasets.

## Results

The YH study was funded in March 2021. Data collection for this project began in April 2022. Enrollment concluded in February 2025, with data collection extending through February 2027. Across the 8 recruitment sites, a total of 441,420 invitations were sent, of which 1.6% (n=7114) resulted in a consented participant (Figure 1). The response percentage varied across recruitment sites, ranging from 0.4% to 3.9% with a median of 1.0%. Of consented participants, 6651 (93.5%) completed their baseline questionnaires; these individuals comprise the YH cohort. The YH cohort is 49% (n=3259) female with a mean age of 8.6 (SD 5.5) years. As of December 19, 2025, 42% (n=2794) and 32% (n=2129) of eligible participants have completed their 1- and 2-year e-visits, respectively.

To date, email communication has been the preferred method among participants. Throughout the study period, the study staff has been tracking missed phone calls to identify any recurring concerns and to ensure timely callbacks. Some common inquiries to our team include requests for more information about the study, questions about the study’s validity, requests to amend survey responses post-submission, assistance with the survey link or mobile app, and ensuring the study is available in Spanish.

Deidentified data from the YH study can be made available upon request to the corresponding author after approval by co-investigators in accordance with National Heart, Lung, and Blood Institute policies. Identifiable and other sensitive data (eg, raw EHR data) will only be made available after researchers have provided evidence of the necessary approvals, both from their IRB or comparable entity and from participating health systems.

## Discussion

This ongoing cohort study offers an innovative approach to the collection of CVH measures across developmental stages from infancy through adolescence. By integrating electronic survey instruments with EHR data linkage, the study design harnesses technological advancements to facilitate comprehensive data collection on an unprecedented scale. The YH enrolled sample is very large—almost twice the size of comparable domestic cohorts—and encompasses infants, children, and adolescents from many racial and ethnic backgrounds [42,49]. In particular, almost 8% of enrollees self-identified as multi-racial, potentially allowing for inference in this critical population.

The YH study extends its innovation beyond technological data collection methods to the incorporation of contemporary thematic content in risk factor assessment. The questionnaires capture modern use of digital technologies (screen time) and address current nicotine delivery systems like vaping devices rather than focusing solely on cigarette use. Furthermore, the study contextualizes CVH findings within participants’ social environments by incorporating neighborhood assessments of safety and walkability, as well as evaluations of school experiences, including peer victimization and bullying dynamics.

This study has several limitations. First, study recruitment concentrated in the greater Chicago metropolitan area, limiting generalizability to the broader national pediatric and adolescent populations. Second, data collection for CVH factors relies on the information available in the EHR. Measurement of BMI and BP in-office may not be done according to research best practices, introducing error. Data incompleteness may be prevalent; as our team noted in a prior publication, nonadherence to pediatric screening guidelines is common, resulting in low reporting of laboratory values, namely glucose and cholesterol [49]. Additionally, in this large pediatric population, data capture for the CVH behaviors, including diet, physical activity, nicotine exposure, and sleep, is done via parent- or self-report. Although reports of these data may be subject to measurement error and bias, including recall bias, social desirability bias, and healthy responder bias, using more technologically advanced data capture methods was not logistically or financially possible in this study. Last, study retention is low, but is in line with other web-based cohorts [50]. To combat this issue, we are implementing efforts to retain participants, including reaching out electronically and over the phone to encourage sustained participation.

Despite these limitations, the creation of the YH cohort using pragmatic, technology-enabled data collection methods and big data will elucidate early life CVH trajectories in a modern, highly diverse cohort of children. We plan to develop an actionable CVH growth curve tool for the monitoring of CVH by pediatricians throughout infancy, childhood, and adolescence. YH has the potential to be instrumental in providing foundational research to inform the design of future health promotion interventions.

## Supplementary material

10.2196/82619 Multimedia Appendix 1 Supplemental tables.

## Abbreviations

AAP: American Academy of Pediatrics
ACE: adverse childhood experience
BP: blood pressure
CAB: Community Advisory Board
CV: cardiovascular
CVH: cardiovascular health
EHR: electronic health record
i3C: International Childhood Cardiovascular Cohort
IRB: Institutional Review Board
LE8: Life’s Essential Eight
SHAPE: Society of Health and Physical Educators
YH: Young Hearts
