# Supplementary material for: A Large, Diverse, Urban Cardiovascular Health e-Cohort in Childhood and Adolescence: Protocol for the Young Hearts Study
Source: JMIR Res Protoc. 2026 May 21;15:e82619. doi: 10.2196/82619 (PMC13195178; doi:10.2196/82619)
Supplement: Multimedia Appendix 1 [file resprot-v15-e82619-s001.docx]

**Multimedia Appendix** for manuscript titled “Protocol of a Large, Diverse, Urban Cardiovascular Health e-Cohort Study in Childhood and Adolescence: The Young Hearts Study”

**Date:** December 31, 2025

| **Author** |
| --- |
| Lucia C. Petito |
| Rachel Zmora |
| Aashima Chopra |
| Yaojie Wang |
| Darci Phillips |
| Ileah Rios |
| Mark Pletcher |
| Rupali Ghandi |
| Huma Khan |
| Cheryl Lefaiver |
| Amanda Luff |
| Rashmi Narayan |
| Sarah De Ferranti |
| Holly C. Gooding |
| Stephen Daniels |
| Brad Appelhans |
| Karen Lui |
| Francis Alenghat |
| Rachel Caskey |
| Matthew M. Davis |
| Marc B. Rosenman |
| Darwin R. Labarthe |
| Donald M. Lloyd-Jones |
| Amanda M. Perak |
| Norrina B. Allen |

**Table S1.** Scoring of pediatric cardiovascular health based on an adaptation of the American Heart Association’s Life’s Essential 8 framework.

| **Component** | **Definition** |
| --- | --- |
| BMI | Body weight (kg) divided by height squared (m) converted to percentile for age and sex  For ages 24 months or older   \| Points \| Value \| \| --- \| --- \| \| 100 \| 5^th^-<85^th^ percentile \| \| 80 \| <= 5^th^ percentile \| \| 70 \| 85^th^-<95^th^ percentile \| \| 30 \| 95^th^ percentile - <120% of the 95^th^ percentile \| \| 15 \| 120% of the 95^th^ percentile - <140% of the 95^th^ percentile \| \| 0 \| >= 140% of the 95^th^ percentile \|   Percentile defined by CDC  For ages 0-23 months   \| Points \| Value \| \| --- \| --- \| \| 100 \| < 97.7 percentile \| \| 80 \| <=5^th^ percentile \| \| 75 \| 97.7 – 99.7 percentile \| \| 50 \| >99.7 percentile \|   Percentile defined by WHO |
| Sleep | Ages 6y-18y: Average hours of sleep per night  Ages 4mo-5y: Average hours of sleep per 24h  Used a weighted average to combine reported average hours of sleep on weekdays (5/7) versus weekends (2/7).   \| Points \| Value \| \| --- \| --- \| \| 100 \| Age-appropriate optimal range \| \| 90 \| <1 hour above optimal range \| \| 70 \| <1 below optimal range \| \| 40 \| 1-<2h below or >=1h above optimal range \| \| 20 \| 2-<3h below optimal range \| \| 0 \| >=3h below optimal range \|   For children, age-appropriate optimal sleep ranges were defined as:   - Age 4 to 12 months, 12 to 16 hours per 24 hours (includes naps) - Age 1 to 2 years, 11 to 14 hours per 24 hours - Age 3 to 5 years, 10 to 13 hours per 24 hours - Age 6 to 12 years, 9 to 12 hours - Age 13 to 18 years, 8 to 10 hours   Not defined for infants ages 0-3mo |
| Nicotine exposure | Combustible tobacco use or inhaled nicotine (e.g. e-cigarette) use at any age; or secondhand smoke exposure   \| Points \| Value \| \| --- \| --- \| \| 100 \| Never tried \| \| 50 \| Tried any nicotine product, but >30d ago \| \| 25 \| Currently using inhaled NDS \| \| 0 \| Current combustible use (any within 30d) \|   Subtract 20 points (unless score is 0) for living with active indoor smoker in home |
| Physical activity | For children ages 6-19y: Minutes of moderate- (or greater) intensity activity per week.   \| Points \| Value \| \| --- \| --- \| \| 100 \| >=420 minutes \| \| 90 \| 360-419 \| \| 80 \| 300-359 \| \| 60 \| 240-299 \| \| 40 \| 120-239 \| \| 20 \| 1-119 \| \| 0 \| 0 \|   For children ages 1-5y: 7-day average of minutes of active play per day. Reported as a total of free play time plus organized game time.   \| Points \| Value \| \| --- \| --- \| \| 100 \| >=180 minutes \| \| 90 \| 150-179 \| \| 80 \| 120-149 \| \| 70 \| 90-119 \| \| 50 \| 60-89 \| \| 25 \| 1-59 \| \| 0 \| 0 \|   Not scored in infants ages 0-11mo |
| Screen time | Screen time in minutes calculated as a weighted average of screen time per weekday (5/7) and per weekend (2/7)  Age <24mo:   \| Points \| Value \| \| --- \| --- \| \| 100 \| 0 minutes \| \| 70 \| 1-29 \| \| 40 \| 30-59 \| \| 20 \| 60-119 \| \| 0 \| 120+ \|   Age 2-5y:   \| Points \| Value \| \| --- \| --- \| \| 100 \| <60 minutes \| \| 70 \| 60-89 \| \| 40 \| 90-119 \| \| 20 \| 120-179 \| \| 0 \| 180+ \|   Age 6+y:   \| Points \| Value \| \| --- \| --- \| \| 100 \| <120 minutes \| \| 70 \| 120-149 \| \| 40 \| 150-179 \| \| 20 \| 180-239 \| \| 0 \| 240+ \| |
| Diet | **For ages <12mo**   \|  \| Age <12mo \| \| --- \| --- \| \| 100 \| breastmilk + started solids at 5 months or older \| \| 70 \| mix of breastmilk + formula + started solids at 5 months or older \| \| 60 \| formula only + started solids at 5 months or older \| \| 50 \| breastmilk only but started solids at <5 months \| \| 30 \| if any formula and started solids at <5 months \| \| 0 \| not given \|   * Missing solids start date IF child <12mo assumed to mean that the child has not yet started solids  **For ages 1-18y**  Scoring system created from an abbreviated MEPA.  Each point below is worth 100/6 (18.xx) points. Points were totaled across all 5 food categories. Higher score implies better diet.   \|  \| **Age 1-<2y** \| **Age 2-5y** \| **Age 6-11y** \| **Age 12-17** \| \| --- \| --- \| --- \| --- \| --- \| \| **Food** \| **Answer to score 1 point** \| \| \| \| \| Fruits/day \| ≥1 s/d \| ≥1 s/d \| ≥1 s/d \| ≥1 s/d \| \| Veg/day \| ≥3 s/d \| ≥2 s/d \| ≥3 s/d \| ≥5 s/d \| \| Fast food \| Never or rarely \| Never or rarely OR <1/wk \| Never or rarely OR <1/wk \| Never or rarely OR <1/wk \| \| Salty packaged \| Never or rarely \| Never or rarely OR <1/wk \| Never or rarely OR <1/wk \| Never or rarely OR <1/wk \| \|  \| **Answers on BOTH SSB & Sweets needed to score 2 points (vs 0)** \| \| \| \| \| SSB/Sweets \| Doesn’t drink SSB AND Sweets never or rarely \| ADD SSB/wk + sweets/wk, total <=3 per week \| ADD SSB/wk + sweets/wk, total <=3 per week \| ADD SSB/wk + sweets/wk, total <=4 per week \|   Abbreviations. s/d = servings/day; wk = week; y = year; SSB = sugar sweetened beverage  Note – serving size for fruits and vegetables was asked using a developmentally appropriate reference size. |

**Table S2.** Scales and subscales and their ranges collected through surveys at e-visits: the Young Hearts Study.

| **Scale** | **Subscale** | **Score range** |
| --- | --- | --- |
| Strengths and Difficulties (SDQ) Questionnaire | Emotional symptoms | 0-10 |
|  | Conduct problems | 0-10 |
|  | Hyperactivity/inattention | 0-10 |
|  | Peer relationship problems | 0-10 |
|  | Prosocial behavior | 0-10 |
|  | Total difficulties | 0-40 |
| School Experiences | School | 0-16 |
|  | Peers | 0-16 |
| Perceived Stress Scale (PSS-4) | --- | 0-16 |
| Patient Health Questionnaire (PHQ-2) | --- | 0-6 |
| Diet Behavior and Nutrition (DBQ) | --- | --- |
| Comprehensive Feeding Practices Questionnaire subset | Emotional Regulation | 0-3 |
|  | Food as a Reward | 0-2 |
| Neighborhood Questionnaire | Neighborhood Safety | --- |
|  | Social Involvement | --- |
|  | Public Services | --- |
| Six-Item Food Security Scale | --- | 0-6 |
| Financial Strain | --- | 2-8 |
| Adverse Childhood Experiences Questionnaire (ACE-Q) | Traditional ACEs | 0-10 |
|  | Additional ACEs | 0-7 |
|  | Total ACEs | 0-17 |

**Table S3.** Sample size needed to achieve a particular 95% confidence interval width around a particular quantile assuming a standard normal distribution: the Young Hearts study.

| **Width of 95% CI** | **Sample Size** | | |
| --- | --- | --- | --- |
|  | *Median (Z=0)* | *16^th^ percentile (Z=-1)* | *2.5^th^ percentile (Z=-2)* |
| 0.10 | 1 540 | 2 300 | 4 492 |
| 0.12 | 1 070 | 1 598 | 3 121 |
| 0.14 | 787 | 1 175 | 2 294 |
| 0.16 | 603 | 901 | 1 757 |
| 0.18 | 477 | 712 | 1 389 |
| 0.20 | 387 | 578 | 1 126 |
| 0.22 | 320 | 478 | 932 |
| 0.24 | 270 | 402 | 783 |
| 0.26 | 230 | 343 | 668 |
| 0.28 | 199 | 296 | 577 |
| 0.30 | 174 | 259 | 503 |
